# Liquid crystal thermography for breast cancer detection: principles, current evidence, limitations, and future directions

**DOI:** 10.1186/s43046-026-00383-6

**Published:** 2026-07-15

**Authors:** Ainnur Bazilah Mohd Sha’ari, Heba Mohammed Arafat, Tengku Ahmad Damitri Al Astani Tengku Din, Bazli Md Yusoff, Ohood Mohammed Shamallakh, Wan Faiziah Wan Abdul Rahman

**Affiliations:** 1 Department of Pathology, School of Medical Sciences, Universiti Sains Malaysia, Health Campus, Kubang Kerian, Kelantan 16150 Malaysia; 2 Department of Chemical Pathology, School of Medical Sciences, Universiti Sains Malaysia, Health Campus, Kubang Kerian, Kelantan 16150 Malaysia; 3 Department of Radiology, School of Medical Sciences, Universiti Sains Malaysia, Health Campus, Kubang Kerian, Kelantan 16150 Malaysia; 4 Department of Medical Laboratory Sciences, Faculty of Health Sciences, Islamic University of Gaza, Gaza, Palestinian Territory

**Keywords:** Liquid crystal thermography, Breast cancer detection, Early detection, Non-invasive screening, Thermochromic materials, Braster

## Abstract

Breast cancer remains a leading cause of cancer-related mortality in women worldwide, making early and accurate detection a global clinical priority. Conventional screening modalities, including mammography, ultrasonography, and Magnetic Resonance Imaging **(**MRI), each have well-established strengths and limitations; in particular, mammographic sensitivity is reduced in women with heterogeneously or extremely dense breast tissue, where it may fall to approximately 50%. Liquid Crystal Thermography (LCT) is a contact-based, non-invasive, radiation-free technique that uses thermochromic liquid crystal (TLC) foils to map surface thermal patterns on the breast and has been investigated as a potential adjunct to standard breast screening. This narrative review summarises the physical and physiological basis of LCT, describes the Braster-based contact thermography workflow, evaluates published diagnostic performance data, and provides a structured analysis of current limitations, confounding factors, and evidence gaps in comparison with established screening modalities. Available studies suggest that LCT may have a role as an investigational adjunctive or triage tool, particularly in settings with limited access to standard imaging; however, the evidence base is heterogeneous, predominantly small in sample size, and methodologically variable, and is insufficient at this stage to establish LCT as a standalone substitute for mammography. The U.S. Food and Drug Administration (FDA) has explicitly stated that thermography should not be used in place of mammography for breast cancer screening or diagnosis. Any potential advantage of LCT in dense breast tissue remains unconfirmed, as no published comparative study has yet provided head-to-head false-negative and false-positive counts across modalities within this subgroup. Preliminary observational data from an ongoing Malaysian case series are included only to illustrate the clinical workflow; these findings are exploratory, unpublished, and not peer-reviewed, and should not be interpreted as confirmatory evidence of diagnostic performance. Future research priorities include large-scale prospective validation, standardised acquisition and interpretation protocols, and rigorous comparative reporting of sensitivity, specificity, and misclassification rates.

## Highlights

Summarizes clinical evidence for liquid crystal thermography in breast screening.

Critically evaluates dense-breast performance claims in the context of available evidence.

Identifies sources of bias, confounders, and gaps limiting current accuracy claims.

Proposes a standardized LCT acquisition/interpretation workflow for reproducibility.

Outlines future directions: AI pattern analysis and large prospective trials.

## Introduction

Breast cancer is the most commonly diagnosed cancer and the leading cause of cancer-related mortality in women worldwide, with an estimated 2.3 million new cases and over 670,000 deaths recorded in 2022 [1]. It arises from the uncontrolled proliferation of mammary epithelial cells and frequently progresses without clinical symptoms in its early stages, often resulting in diagnosis at an advanced and less treatable stage [2]. In low- and middle-income countries, where screening infrastructure is limited, a substantial proportion of cases present with late-stage or metastatic disease, a pattern that contributes directly to the persistently high breast cancer mortality burden in these settings [3]. Factors including limited public awareness, inadequate healthcare access, low screening uptake, and the absence of organized population-based screening programmes each contribute to diagnostic delay. Early and accurate detection remains the single most modifiable determinant of breast cancer survival: five-year survival rates exceed 90% in high-income countries with established screening programmes, compared with approximately 66% in India and 40% in South Africa, disparities attributable in large part to stage at diagnosis [4].

Traditional screening methods, including Breast Self-Examination (BSE), Clinical Breast Examination (CBE), and mammography, remain the clinical standard, but each carries recognized limitations [5–7]. Mammography, the gold standard for breast cancer screening, has reduced sensitivity in women with dense breast tissue, detecting approximately 50% of cancers in this subgroup compared with 70–80% in women with fatty tissue [8]. Ultrasonography is operator-dependent and unable to reliably detect microcalcifications, which appear in up to 51% of malignant non-mass breast findings [9]. Magnetic Resonance Imaging (MRI), while highly sensitive, carries a false-positive rate reported between 6 and 41% depending on the study setting and population, is resource-intensive, and is not widely accessible in most health systems [10]. The incidence of breast cancer is rising among premenopausal women, many of whom have dense breast tissue, which both increases cancer risk and reduces the effectiveness of conventional imaging [8, 11]. These clinical limitations have motivated research into adjunctive and complementary technologies, including thermographic approaches [12, 13].

It is also important to recognize that thermographic and liquid crystal thermographic (LCT) approaches have significant, well-documented limitations [12–14]. These include an inability to detect microcalcifications or provide anatomical lesion localization; microcalcifications are detectable by mammography and not by surface thermal mapping, as the thermographic signal reflects vascular activity rather than structural tissue composition [12]. Susceptibility to false-positive findings from benign inflammatory processes such as mastitis, uncertain sensitivity for small or non-angiogenic malignancies, and limited depth penetration are each mechanistically inherent to surface thermal assessment and cannot be resolved through image processing alone [12, 15, 16]. Device-specific confounders, including ambient temperature, foil contact pressure, patient menstrual phase, and recent physical activity, further affect reproducibility and remain incompletely controlled in the existing literature [17]. Historical liquid crystal contact thermography (LCT), first described by Davison et al. in 1972 and subsequently evaluated through the 1980s and 1990s, demonstrated these same fundamental constraints, including reduced detection rates for smaller lesions [18–20]. These limitations must therefore be weighed alongside those of conventional modalities in any clinical appraisal [13].

A critical regulatory consideration must also be established at the outset. The U.S. Food and Drug Administration (FDA) has explicitly stated that thermography should not be used in place of mammography to screen for or diagnose breast cancer and is not effective as a standalone screening tool for breast cancer [21]. This position reflects the absence of high-quality prospective evidence demonstrating that thermography, including contact LCT, can reliably replace mammography in any screening context [13, 14]. Accordingly, throughout this review, LCT is framed exclusively as an investigational adjunctive or triage modality under evaluation, and not as a primary screening substitute for mammography [17, 21]. Any use of LCT in clinical research settings should be understood within this regulatory and evidentiary framework [21].

While several narrative and systematic reviews of thermography in breast cancer have been published, the present work is distinguished from prior literature in five respects. First, it focuses specifically on contact-based liquid crystal thermography and the Braster platform, rather than addressing non-contact infrared thermography broadly [12, 13]. Second, it provides a structured, bidirectional comparative analysis against each conventional modality, explicitly addressing both what LCT cannot do relative to established methods and where established methods are limited. Third, it includes a QUADAS-2 domain-level quality appraisal of all included diagnostic performance studies. Fourth, it provides a detailed analysis of Braster-specific colorimetric calibration requirements and artefact sources, including illumination stability, camera white balance, foil batch variability, and ambient temperature drift, specific to the contact colorimetric design and not addressed in prior thermography reviews. Fifth, it integrates the full regulatory framework, including the FDA’s explicit “no substitute” position and the historical LCT limitations literature from 1972 to 1991, to situate contemporary Braster evidence within an appropriate long-term evidentiary context and guard against repeating historical optimism without validated incremental improvement [18–21].

This review explores the physical principles of LCT, the Braster-based workflow, available diagnostic performance data, a structured comparative analysis against conventional modalities, and key limitations and future research directions, with the aim of providing a clinically realistic and balanced appraisal of where LCT currently stands as an investigational tool in breast cancer detection.

## Methods

### Review design and type

This article is a narrative review of the current evidence on LCT for breast cancer detection. It does not constitute a systematic review, and no formal review protocol was prospectively registered. A PRISMA-guided search and selection process was not applied; however, to maximize transparency and reduce the risk of selective citation, the literature identification process is described below, and the evidence is evaluated with explicit reference to study design, sample size, reference standard, population characteristics, and key methodological limitations for each included study.

The decision to conduct a narrative rather than a systematic review reflects the substantial methodological heterogeneity across thermography studies, which differ in device type, acquisition protocol, patient population, reference standard, and outcome reporting. This heterogeneity renders formal quantitative pooling unreliable, as confirmed by the 2024 systematic review and meta-analysis by Goni-Arana et al., which reported an I² of 99.1% for specificity across included non-contact infrared thermography studies, indicating that pooled performance estimates are not meaningful [13]. A narrative approach is therefore appropriate for synthesising an emerging and technically heterogeneous field while identifying critical evidence gaps.

### Literature search strategy

Literature was identified through structured searches of PubMed/MEDLINE and Google Scholar, supplemented by manual reference list searching of relevant review articles and key primary studies. Searches were conducted between August and December 2025 and covered publications from 1970 to 2025. No formal language restriction was applied; however, only English-language publications were identified within the search results and were subsequently included in the final review. No non-English-language studies meeting the inclusion criteria were identified.

The following keyword combinations were used in the search strategy: LCT OR contact thermography breast; Braster device OR liquid crystal contact thermography; infrared thermography breast cancer; thermochromic liquid crystals breast screening; breast cancer dense tissue thermography; thermography sensitivity specificity breast; and LCT false-positive OR false-negative breast cancer.

Historical publications from the 1970–1990 s were specifically sought to contextualise the origins and early clinical limitations of liquid crystal contact thermography, given the recognized importance of historical evidence for situating contemporary device performance claims in appropriate context [18–20].

#### Inclusion criteria

Studies reporting diagnostic performance data (sensitivity and/or specificity) for thermography or LCT in breast cancer detection; studies describing clinical workflow, device specifications, or technical methodology for contact or infrared thermography; and review articles and meta-analyses reporting synthesised evidence on thermographic breast screening.

#### Exclusion criteria

Studies using thermography exclusively for non-breast applications; conference abstracts without accessible full text; studies with no identifiable reference standard; animal or phantom studies.

No formal PRISMA flow diagram is provided, consistent with the narrative review design. This limitation is acknowledged, and a future systematic review with PRISMA reporting and QUADAS-2 quality appraisal is recommended as a research priority.

### Evidence quality appraisal

Given that this is a narrative review, a formal QUADAS-2 appraisal was not prospectively registered. However, to reduce selective citation bias and to make the methodological limitations of the included diagnostic studies explicit, a retrospective QUADAS-2 domain-level appraisal was undertaken for all nine studies across four standard domains: Patient Selection (D1), Index Test (D2), Reference Standard (D3), and Flow and Timing (D4), with risk of bias and applicability concerns rated as low, high, or unclear in accordance with the QUADAS-2 framework [22]. The results are presented in Table 1.

The dominant source of methodological risk was patient selection bias. All studies were conducted in symptomatic, referred, biopsy-enriched, or cancer-enriched populations rather than true consecutive screening cohorts, meaning that performance figures cannot be generalised to the community screening settings for which Braster LCT is primarily intended [22]. Index test bias arose in studies using post-hoc threshold optimisation, single-dataset machine-learning validation, or selective reporting of multiple interpretation methods. A cross-applicability concern further affects all eight non-Braster studies, as non-contact infrared thermography platforms and AI-based algorithms differ fundamentally from the Braster RGB colorimetric contact mechanism; sensitivity and specificity reported for these devices cannot be assumed to apply to Braster LCT [13, 23].

Reference standard quality was generally stronger, with six of nine studies using biopsy histopathology as the primary reference standard (Table 1). However, three studies substituted imaging follow-up for biopsy in test-negative cases, introducing verification bias that inflates apparent sensitivity and underestimates false-negative rates [24–26]. Flow and timing concerns were rated high or unclear in all but one study, consistent with the extreme between-study heterogeneity in specificity (I² = 99.1%) reported by Goni-Arana et al. (2024) [13], confirming that pooled estimates are not clinically meaningful.

**Table 1:** QUADAS-2 domain-level quality appraisal of included diagnostic performance studies

Study	Patient selection (RoB)	Index test (RoB)	Reference standard (RoB)	Flow and timing (RoB)	Patient selection (Applicability)	Index test (Applicability)	Reference standard (Applicability)
Hodorowicz-Zaniewska et al. (2020) [17]	High	Unclear	Low	High	High	Low	Low
Singh et al., 2021 [27]	High	High	High	High	High	High	Moderate
Da Luz et al. (2020) [25]	High	High	Low	High	High	High	Low
Alikhassi et al. (2018) [24]	High	High	High	High	High	High	Moderate
Sarigoz et al. (2018) [28]	High	Unclear	Low	Moderate	High	Low	Low
Prasad et al. (2016) [29]	High	High	Low	High	High	Low	Low
Acharya et al. (2012) [30]	High	High	Moderate	High	High	High	Moderate
Wishart et al. (2010) [26]	High	Unclear	Low	High	High	Moderate	Low
Parisky et al. (2003) [15]	High	High	Low	High	High	High	Low

RoB = risk of bias. Ratings were assigned retrospectively in accordance with the QUADAS-2 framework [22]

Table 1 is therefore presented as a structured descriptive appraisal and should not be used as a basis for evidence pooling or comparative ranking. Future research should incorporate QUADAS-2 prospectively, with pre-specified signalling questions and independent dual rating, to enable valid evidence synthesis across thermography modalities.

## Conventional screening techniques

Conventional breast cancer screening relies primarily on clinical examination and imaging-based modalities, each with distinct strengths and limitations [7, 31]. Breast self-examination and clinical breast examination remain relevant in selected settings, while mammography, ultrasonography, and MRI form the core of modern screening and diagnostic pathways [7, 32]. In practice, the choice of modality depends on age, breast density, risk status, clinical presentation, and resource availability [6, 8].

### Breast self-examination and clinical breast examination

Breast self-examination (BSE) and clinical breast examination (CBE) are simple, non-invasive methods used to detect breast abnormalities through visual inspection and palpation [5, 33]. BSE may improve breast awareness and prompt earlier medical evaluation when changes are noticed, but it has limited sensitivity and should not be viewed as a substitute for imaging-based screening [5, 6]. CBE, when performed by trained clinicians, can help identify palpable masses and is particularly useful in settings where access to imaging is limited, but its diagnostic performance is modest and operator-dependent [33, 34].

### Mammography

Mammography remains the gold standard for population-level breast cancer screening and is the only widely used modality shown to reduce breast cancer mortality in screening trials [7, 35]. Its major strength is the detection of microcalcifications and structural abnormalities that may represent early ductal carcinoma in situ or invasive disease [7, 32]. Mammography is also widely available, relatively low cost, and well established in screening guidelines for average-risk women [31, 35].

Its main limitations are reduced sensitivity in women with dense breast tissue, discomfort due to breast compression, and exposure to ionising radiation [8, 36]. Because dense fibroglandular tissue can obscure lesions, supplemental imaging with ultrasound or MRI is often considered in selected women at increased risk or with dense breasts [8, 32].

### Breast ultrasonography

Breast ultrasonography is a non-invasive imaging technique that uses high-frequency sound waves to evaluate breast tissue in real time [9, 10]. It is especially useful for characterising palpable masses, distinguishing cystic from solid lesions, evaluating dense breasts, and guiding biopsy procedures [9, 37]. Unlike mammography, ultrasound does not involve ionising radiation and can be used safely in younger patients and pregnant women [9].

However, ultrasound is operator-dependent, may generate false-positive findings, and is less effective at detecting microcalcifications than mammography [9, 10]. For this reason, it is generally used as a complementary rather than a standalone screening modality, particularly in women with dense breasts or equivocal mammographic findings [7, 8].

### Magnetic resonance imaging

Breast MRI is the most sensitive imaging modality for detecting breast cancer and is particularly valuable in women at high lifetime risk, including those with hereditary predisposition or strong family history [10, 38]. It provides detailed three-dimensional anatomical information and is highly effective for detecting multifocal, multicentric, and contralateral disease [32, 38]. MRI is therefore widely used as a supplemental screening tool in high-risk populations and in selected diagnostic situations [7, 38].

Despite its high sensitivity, MRI is costly, less accessible, time-consuming, and associated with a false-positive rate reported between 6 and 41% depending on the study population and setting, and is more costly and less accessible than mammography [10, 38]. It also requires intravenous contrast in most screening applications, which adds cost, complexity, and the possibility of contrast-related adverse effects [32, 38]. As a result, MRI is reserved for women at elevated risk or for specific diagnostic indications rather than routine population-level screening [7, 31].

## Liquid crystal thermography

LCT is a contact-based thermographic technique that uses thermochromic liquid crystals to map temperature-related colour changes on the breast surface [17]. It is based on the premise that malignancy-associated angiogenesis and altered perfusion may produce localised thermal asymmetry, which can be detected as a visible colour pattern [13, 17]. Unlike conventional infrared thermography, LCT uses direct contact foils rather than a non-contact infrared camera, and the resulting colourimetric signal is then interpreted visually or with computer-assisted analysis [17, 23].

### Thermography and liquid crystal principles

Breast thermography is a physiological imaging method that evaluates heat patterns rather than breast structure [12]. The underlying concept is that malignant tissue may alter local blood flow, metabolism, and vascular permeability, leading to surface temperature differences that can be detected at the skin level [12, 14]. This makes thermography fundamentally different from mammography, ultrasound, and MRI, which are structural or functional imaging techniques that directly visualise tissue architecture [39].

LCT extends this idea by using TLC foils that change colour within narrow temperature ranges [23]. When placed in contact with the skin and illuminated with white light, the foil reflects specific wavelengths depending on local temperature, producing a visible thermal map [23]. In principle, this approach may offer a portable and non-invasive way to assess breast thermal patterns, but its clinical value remains dependent on accurate acquisition, calibration, and interpretation [17, 23].

### Historical development

Breast thermography has a long history, beginning with early observations in the mid-twentieth century that breast malignancies could be associated with abnormal surface temperature patterns [18, 19]. The method attracted interest because it was non-invasive, painless, and free of ionising radiation [18]. However, its early clinical use was limited by poor standardisation, variable interpretation, and inconsistent diagnostic performance [12, 13].

The FDA later cleared thermography only as an adjunctive tool and has repeatedly stated that it should not be used in place of mammography for breast cancer screening or diagnosis. This regulatory position reflects the lack of evidence supporting thermography as a standalone screening method and remains central to how LCT should be interpreted in clinical research [21]. Contemporary LCT therefore represents a modern contact-based refinement of an older physiologic concept rather than a replacement for established imaging [17, 23].

### Mechanism of thermochromic liquid crystals

Thermochromic liquid crystals belong to the chiral nematic phase, in which molecules are arranged in a helical structure that reflects specific wavelengths of light. As temperature changes, the helix pitch changes, shifting the reflected wavelength and producing a visible colour transition [23, 40]. This property makes TLCs useful for mapping small surface temperature differences because a narrow thermal range can correspond to a distinct colour band [23, 40].

To improve durability and stability, TLCs are commonly microencapsulated. Microencapsulation protects the crystal material from moisture, handling damage, and environmental degradation while preserving the colour-response behaviour needed for diagnostic use [40]. In LCT systems, this colour response must remain stable and reproducible across examinations, because any drift in the temperature-to-colour relationship can affect the reliability of interpretation [23, 40].

### Braster device and clinical workflow

The Braster system is a Class IIa medical device designed for contact LCT and is one of the best-known commercial platforms in this field [23]. It uses a sequence of thermochromic foils placed directly on the breast surface to generate colourimetric images that represent local surface temperature patterns [17, 23]. The workflow typically begins with patient acclimatisation in a controlled room environment, followed by sequential application of foils to cover the breast surface, and finally RGB image acquisition for later interpretation by a human reader or AI-based system [23].

**Fig. 1:**
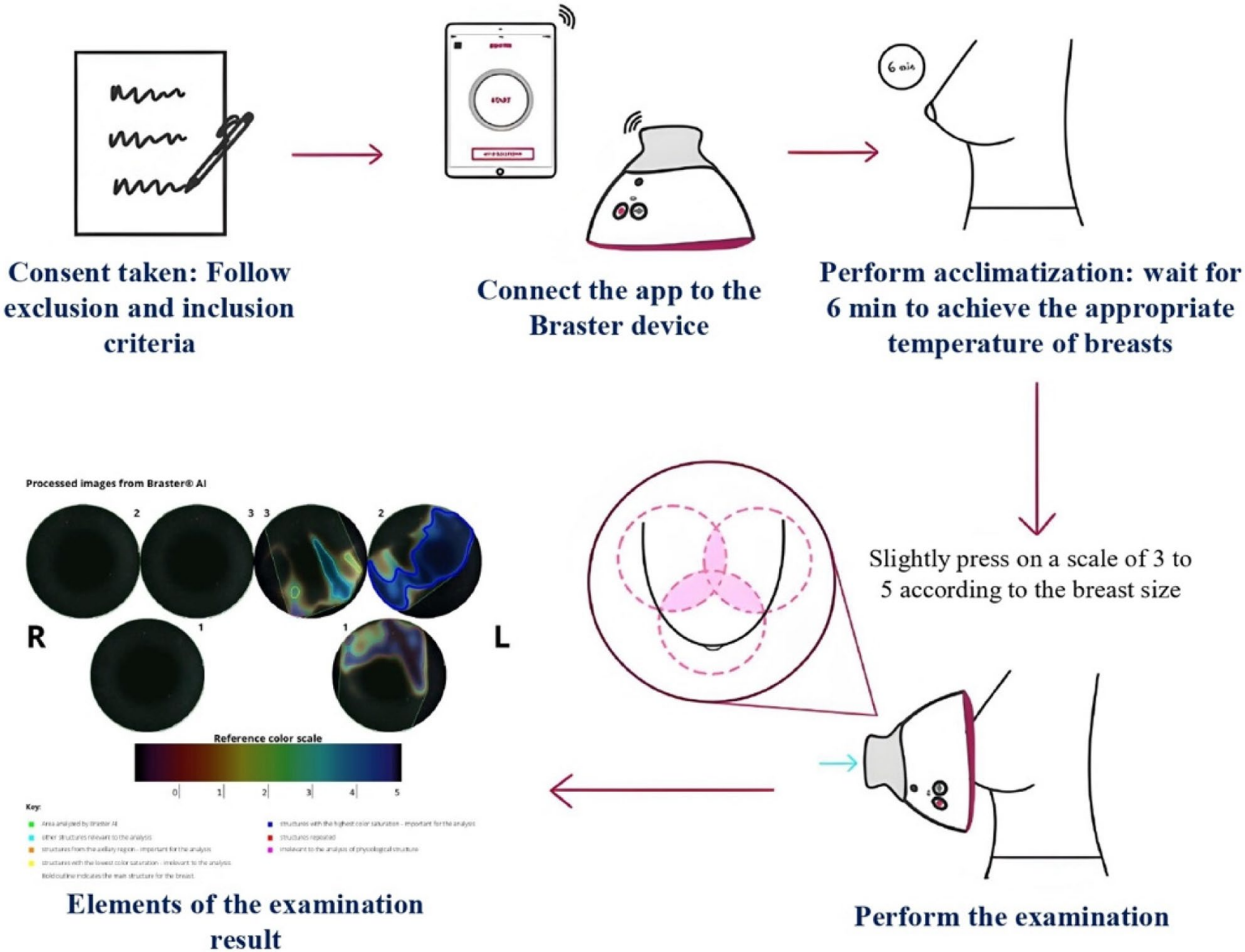
Schematic workflow of the Braster-based liquid crystal thermography procedure. The examination comprises three stages: patient acclimatization, sequential application of thermochromic liquid crystal foils to the breast surface, and RGB image acquisition for subsequent human or AI-based interpretation

Three foil types are used to cover overlapping temperature ranges, allowing the system to capture variation across different breast surfaces and patient baseline temperatures [23]. The sequential nature of the protocol means that examination quality depends on consistent foil placement, stable room conditions, and accurate image capture [17]. In practice, this makes the procedure more structured than conventional infrared thermography, but also more sensitive to acquisition-related variability [13, 17]. The three-stage Braster LCT examination procedure, comprising patient acclimatization, sequential thermochromic foil application, and RGB image acquisition, is illustrated in Fig. 1.

### Key practical advantages and limitations

LCT has several practical advantages. It is non-invasive, does not use ionising radiation, and may be more comfortable than compression-based imaging [21]. Its portability and relatively low equipment requirements make it potentially useful in low-resource settings, where access to mammography or MRI may be limited [14, 41]. These features have made LCT attractive as a possible adjunctive or triage tool, especially in populations where conventional imaging is less accessible [17].

At the same time, LCT has important limitations. It cannot localise lesions anatomically, cannot detect microcalcifications, and cannot reliably distinguish benign from malignant causes of thermal asymmetry [12, 15]. Its diagnostic performance is also sensitive to ambient temperature, foil, handling, contact pressure, and other technical variables that can alter colour interpretation [17, 23]. For these reasons, LCT should be regarded as an investigational physiologic imaging method rather than a substitute for established breast imaging modalities [21].

## Methodological considerations

### Braster device: colorimetric calibration and technical constraints

The Braster system acquires diagnostic information through RGB colorimetric mapping of TLC foils placed in direct contact with the breast surface. Each foil reflects specific wavelengths of polychromatic white light depending on local surface temperature, producing a colour pattern that encodes the thermal distribution across the breast. Because the entire diagnostic signal depends on accurate colour-to-temperature correspondence, colorimetric calibration is a central technical requirement that must be explicitly understood when interpreting Braster-derived data.

Three foil types are used in sequence, spanning overlapping temperature ranges: Foil 1 (31.5–33.1 °C), Foil 2 (32.8–34.4 °C), and Foil 3 (34.1–35.7 °C). The overlapping ranges are designed to accommodate inter-individual variation in baseline breast surface temperature. However, accurate colorimetric interpretation depends on the following conditions being controlled or verified at the time of each examination [17, 23].

- Illumination stability: The polychromatic white light source must maintain consistent spectral output across all foil applications. Variation in light intensity or colour temperature between applications introduces systematic colour-mapping error that cannot be distinguished from a true thermal asymmetry.
- Camera white balance: White balance must be fixed and calibrated to the light source before each examination session. Automatic white balance adjustment — which may occur between sequential image captures — can alter the apparent colour of a foil at the same true temperature, introducing false asymmetry.
- Foil batch variability: TLC foils are manufactured to defined colour-response specifications, but batch-to-batch variability in the polymer matrix or microencapsulation process can shift the colour response curve within the designated temperature range. No published standardised batch verification protocol for the Braster foils has been identified in the reviewed literature.
- Ambient temperature drift: Each foil application involves a fresh foil placed on a different breast zone sequentially. If ambient room temperature fluctuates between applications, the thermal equilibration baseline for each zone differs, potentially generating artificial asymmetry between early and late acquisitions in the same session.

These factors represent recognised sources of systematic and random error in LCT colorimetric data. The current published Braster literature does not report standardised controls for all of these variables, and this represents a significant methodological gap that limits the reproducibility and comparability of results across studies and clinical sites. Future standardisation work should define required illumination specifications, camera calibration procedures, foil batch acceptance criteria, and permitted ambient temperature range for valid examinations [17, 42]. As shown in Fig. 1, each foil is applied sequentially to a different breast zone; ambient temperature stability during this sequential process is therefore essential to avoid thermal drift between acquisitions [17, 23].

### Confounder control in LCT acquisition

The reproducibility of LCT findings is substantially dependent on control of physiological and environmental confounders that can alter breast surface temperature independently of any malignant process. The confounders summarised in Table 2 must be explicitly acknowledged and, where possible, controlled in clinical and research applications of LCT [12, 17, 43].

**Table 2:** Physiological and environmental confounders affecting LCT acquisition, their mechanistic effects on breast surface thermal readings, and recommended protocol-level control measures

Confounder	Effect on LCT	Recommended Control
Ambient room temperature	Higher ambient temperature reduces heat dissipation from the breast, artificially elevating surface readings	Maintain examination room at 18–24 °C and document room temperature for each session.
Ambient humidity	High humidity reduces evaporative cooling, elevating surface thermal readings	Document and standardize examination room humidity
Recent physical activity	Exercise increases peripheral blood flow and bilateral surface temperature.	Instruct patients to avoid exercise for at least 2 h before examination.
Menstrual cycle phase	Breast vascularity and thermal patterns vary across the cycle; the luteal phase may produce greater thermal asymmetry.	Ideally perform examinations in days 5–12 of the menstrual cycle in premenopausal women and document cycle phase.
Mastitis, breast abscess, or active inflammation	Localised thermal elevation may mimic malignancy-related angiogenesis on thermal imaging.	Document active inflammatory breast conditions, interpret findings cautiously, and recommend follow-up imaging when needed.
Recent breast surgery or radiation	Post-surgical and post-radiation inflammatory changes alter local vascular patterns	Defer examination for at least 3–6 months after treatment and document surgical history.
Lactation	Lactation markedly alters breast perfusion and surface thermal patterns.	LCT not recommended during active lactation
Contact pressure during foil placement	Variable pressure can compress superficial vasculature, reduce surface thermal emission, and create artefactual asymmetry.	Use a standardised minimal-pressure protocol and trained operators.
Foil-to-skin contact duration	Incomplete equilibration may underestimate true local temperature if contact time is too short.	Use a standardised 15-second minimum contact duration with timer verification.

Without explicit protocol-level controls for each of these variables, and their documentation per participant, the false-positive rate in LCT studies will remain difficult to interpret and is expected to remain elevated, consistent with the well-known false-positive problem in thermography identified across the literature, including specificity of 44% reported by Arora et al. [16] and 51% by Parisky et al. [15] in post-mammography selected populations. Existing published Braster studies do not uniformly report confounder control protocols, which represents an important limitation in interpreting their reported performance figures.

### Acclimatisation protocol and evidence base

The Braster examination workflow (Fig. 1) requires a patient acclimatisation period prior to foil application. A minimum of 15 min of room acclimatisation, during which the patient disrobes and rests in the examination room at a controlled ambient temperature, is consistent with established thermography practice standards, which require that skin surface temperature reaches stable equilibrium with the ambient environment before thermal asymmetries reflecting internal pathology can be reliably distinguished from transient surface variation [17, 23].

The 15-second per-foil contact duration is manufacturer-specified and reflects the time required for each TLC foil to equilibrate to local skin surface temperature. This duration is distinct from the longer room acclimatisation phase and is specific to the contact thermography acquisition mechanism. However, independent validation of this 15-second contact duration under varying ambient conditions and breast surface temperature ranges has not been identified in the published literature. This means the manufacturer-specified time should be treated as a procedural requirement rather than a validated physiological optimum. In clinical practice, foil handling time, patient movement, and ambient fluctuation may mean that full thermal equilibration within the 15-second window is not consistently achieved.

The following minimum protocol requirements, consistent with published thermography acquisition standards, should be adopted and explicitly reported in all future Braster LCT studies [17, 23]. Room temperature maintained at 18–24 °C and documented at the start and end of each session; patient disrobed and rested in the examination room for a minimum of 15 min before any foil application; no physical activity, eating, or topical application to the breast skin on the day of examination; menstrual cycle phase documented for all premenopausal participants; and all sessions conducted by a trained operator following a standardised protocol with a documented foil handling procedure [17, 23].

## Comparative performance of screening modalities

A clinically meaningful evaluation of LCT requires comparison with established breast screening modalities in both directions: what conventional methods do well, and where LCT may offer advantages or remain limited [12, 13]. This section therefore compares LCT with mammography, ultrasound, MRI, and infrared thermography, while maintaining the position that LCT is investigational and not a substitute for standard care. The FDA has explicitly stated that thermography, including contact-based liquid crystal thermography, should not be used in place of mammography to screen for or diagnose breast cancer and is not effective as a standalone screening test. That regulatory position is reflected throughout this section  [21].

### Mammography versus LCT

Mammography remains the gold standard for population-level breast cancer screening and is the only imaging modality demonstrated to reduce breast cancer mortality in randomised controlled trials [7, 35]. Its primary diagnostic strength lies in its ability to detect microcalcifications, small calcium deposits that are a critical early marker of ductal carcinoma in situ (DCIS) and early invasive malignancy, which are entirely invisible to thermographic approaches [12]. Mammography provides structural anatomical information, including lesion size, margins, and spatial location, enabling lesion characterisation and guiding targeted biopsy [7]. Sensitivity in women with fatty breast tissue is approximately 70–80% [8].

Mammography is limited by reduced sensitivity in dense breasts, ionising radiation, the need for breast compression, and reduced accessibility in many low- and middle-income settings [8, 36, 41]. These limitations have motivated interest in adjunctive methods such as LCT, particularly for women with dense breasts [17, 43]. LCT does not require ionising radiation or breast compression and is not affected by breast tissue density in the radiological attenuation sense that limits mammographic sensitivity; however, the thermal background of dense vascularised tissue may still affect the signal-to-noise ratio of any malignancy-associated thermal asymmetry [12, 17]. The only published Braster pilot study reported sensitivity of 82% and specificity of 87% in a community-based, predominantly dense-breast population, suggesting a possible complementary role; however, this was a single small study with partial verification bias and cannot be generalised without prospective confirmation [17].

At the same time, LCT cannot detect microcalcifications, cannot localise lesions anatomically, cannot determine lesion depth or extent, and cannot guide biopsy [12, 14]. It also has uncertain sensitivity for small, deeply situated, non-angiogenic, or slowly growing malignancies that may not generate sufficient surface temperature asymmetry [15, 16]. Specificity remains variable across published studies, and benign inflammatory or post-surgical changes can produce false-positive thermal findings [15, 43]. For these reasons, LCT should be considered only as a potential adjunct to mammography, not as a replacement [21].

### Breast ultrasound versus LCT

Breast ultrasonography provides real-time structural imaging and is able to distinguish cystic from solid lesions, assess palpable abnormalities, and guide needle biopsy [9, 10]. It does not use ionising radiation, making it suitable across age groups, including pregnancy [9]. Ultrasound is particularly useful as a supplemental modality in dense breasts and is widely available in many resource-limited settings, especially when compared with MRI [8]. More recent developments such as automated whole-breast ultrasound and AI-assisted interpretation have improved standardisation and diagnostic performance [9].

Despite these strengths, ultrasound is operator-dependent, can miss microcalcifications, and has a meaningful false-positive rate for benign solid lesions such as fibroadenomas [9, 10]. It is also time-consuming when performed manually over the whole breast and is not used as a standalone population screening test in most guidelines [7, 8]. LCT offers a different kind of workflow: it uses sequential foil application to generate a standardized colorimetric map without probe manipulation, which may be easier to administer in settings without experienced sonographers [17]. Digital transmission and AI-based review may also make LCT attractive as a triage tool in low-resource environments, although these applications remain investigational [14, 43].

However, LCT cannot distinguish cystic from solid lesions, cannot provide biopsy guidance, cannot evaluate lesion vascularity by Doppler, and cannot assess axillary lymph nodes [13, 14]. A false-positive LCT result would still require ultrasound follow-up, so LCT cannot replace structural imaging in most diagnostic pathways [21]. Its role, if validated, would be as a prior-probability triage test rather than a diagnostic substitute [14, 17].

### MRI versus LCT

Breast MRI has the highest sensitivity of the major breast imaging modalities and is recommended as a supplemental screening tool for women at elevated lifetime risk, including BRCA mutation carriers [8, 38]. It provides detailed three-dimensional anatomical information, is highly effective for detecting multifocal and bilateral disease, and can define tumour extent and chest wall or skin involvement, which are important for surgical planning [32, 38]. MRI also provides functional information through gadolinium enhancement, allowing assessment of vascularity and enhancement kinetics [38].

MRI is limited by false-positive findings, high cost, limited availability, the need for specialised equipment and expertise, scan duration, and barriers such as claustrophobia [10, 38]. It also requires gadolinium contrast, which carries a small but real risk of adverse reactions and raises concerns about long-term tissue deposition [38]. LCT offers some practical advantages in this context: it is non-invasive, does not require contrast, involves no ionising radiation or magnetic field, and can be performed in a non-specialist setting within a relatively short examination time [12, 17]. Its portability and lower cost make it potentially useful where MRI infrastructure is absent [14].

These advantages do not make LCT diagnostically equivalent to MRI. LCT measures surface thermal asymmetry and cannot visualise internal tumour morphology, enhancement kinetics, lesion depth, or deep structural involvement [12, 14]. It cannot assess nodal status or chest wall invasion, and it has no validated role as an alternative to MRI in high-risk screening [13, 21]. LCT may eventually be explored as an initial functional layer in settings where MRI is inaccessible, but that role would require prospective validation with biopsy-confirmed outcomes and explicit reporting of false-negative results [13].

### Infrared thermography versus LCT (contact liquid crystal)

Infrared thermography and contact LCT share the same physiological premise: malignancy-associated angiogenesis and vasodilation can produce surface temperature asymmetry that is detectable relative to the contralateral breast [12, 16]. As a result, both modalities share broad diagnostic limitations, including false positives from benign inflammation, false negatives from non-angiogenic or deeply situated lesions, and the absence of anatomical localisation [13, 15]. The main distinction lies in the method of measurement. Infrared thermography uses a non-contact camera to detect emitted thermal radiation, whereas Braster LCT uses direct-contact thermochromic foils that reflect visible light according to local surface temperature [17, 23].

The contact design may reduce some ambient confounders that affect non-contact infrared imaging, including air convection, distance-to-subject variability, and some emissivity-related issues [23]. In principle, this may improve signal stability under variable room conditions. At the same time, direct-contact acquisition introduces contact-pressure artefacts, multi-application thermal drift, foil-handling variability, and batch-to-batch variability in foil colour response [17, 40]. These artefacts are not present in conventional infrared systems and may create false-positive or false-negative results. No published head-to-head study has yet shown that Braster LCT outperforms modern digital infrared thermography under controlled conditions [13, 14].

Recent research has explored alternative portable physiological imaging approaches that seek to overcome the recognised limitations of surface thermography entirely. Bhowmik et al. developed a portable, handheld blood perfusion imager capable of detecting subsurface tumour vascularity in resource-limited settings, demonstrating that direct vascular perfusion measurement may offer improved sensitivity for non-angiogenic or deeply situated lesions that thermographic approaches are known to miss [44]. This highlights that LCT’s dependence on surface thermal emission rather than direct perfusion assessment remains a fundamental constraint, and that overcoming it may ultimately require integration with complementary functional imaging technologies [13, 44].

Published sensitivity and specificity data for thermography are highly heterogeneous, and recent meta-analytic evidence suggests that pooled specificity estimates are not meaningful because of extreme between-study variability (I² = 99.1% for specificity) [13]. The only published prospective Braster study reported a sensitivity of 82% and a specificity of 87%, but it should be interpreted cautiously because of its small size and risk of bias [17]. Early contact thermography studies also identified reduced detection for smaller lesions and substantial false-positive rates, indicating that the historical limitations of surface thermal imaging remain relevant [18–20]. Accordingly, both infrared thermography and LCT should be regarded as investigational adjunctive tools, with LCT’s added value over non-contact infrared thermography still unproven [13, 21].

## Results

### Published diagnostic performance studies

**Fig. 2:**
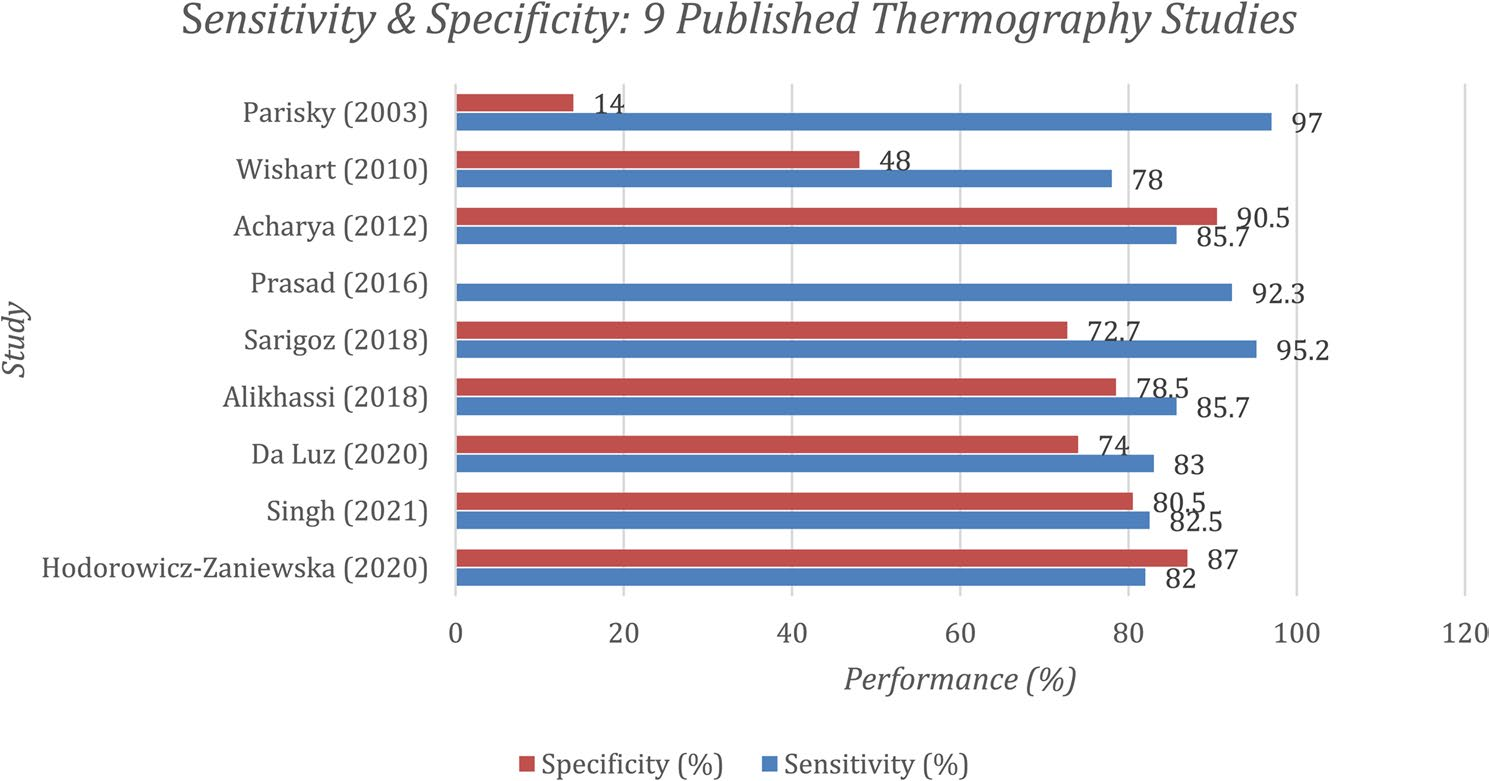
Sensitivity and specificity of published thermography studies. *Note*. Bars are descriptive only; cross-study comparisons are invalid due to heterogeneity in devices, populations, and reference standards. Prasad et al. (2016) specificity was not calculable because the cohort was 100% cancer-enriched and contained no true-negative cases. NR = not reported

Table 3 summarizes nine published diagnostic studies of thermography in breast cancer detection, including one prospective Braster LCT pilot study and eight studies of non-contact infrared or other contact thermography platforms. Sensitivity and specificity values across all nine studies are illustrated in Fig. 2. Across these studies, sensitivity ranged from 78% to 95.2%, while specificity ranged from 14% to 90.5%. However, direct cross-study comparison is not valid because the studies differed in device type, patient population, reference standard, acquisition protocol, and analytical method. Only the Braster pilot study reported a community-screening population with partial breast-density information, and most studies did not report cancer prevalence, stage, or size distribution, 95% confidence intervals, or false-negative and false-positive counts. Accordingly, Table 3 should be read as a structured description of the published evidence base rather than as a pooled estimate of thermography performance.

**Table 3:** Diagnostic performance of thermography studies in breast cancer detection

#	Study	Country	*n*	Device / Type	Population	Breast Density	Reference Standard	Cancer Prevalence	Stage / Size	Acquisition Protocol	Interpretation	Sensitivity % (95% CI)	Specificity % (95% CI)	PPV / NPV	FN / FP	Key Limitation
1	Hodorowicz-Zaniewska et al. (2020) [17]	Poland	274	Braster (contact LCT; Class IIa)	Women ≥ 25 y; prophylactic breast exam; 6 outpatient centers; concurrent US BIRADS 1–5 required	Partial (fatty/mixed/glandular)	Biopsy histopathology (BIRADS ≥4 A); BIRADS 1–2 not biopsied	28.4% (< 50 y); 82.8% (≥ 50 y)	15/53 cancers were T1 (≤ 2 cm); full staging NR	Contact LCT; 3 or 5 foil applications/breast; 15-s contact; clockwise pattern; alcohol abstinence ≥ 2 h; no exertion ≥ 30 min	Two blinded radiologists; third for disagreements; no AI	81.5 (64.1–92.6) < 50 y; 77.8 (67.2–86.2) ≥ 50 y	86.8 (77.2–93.2) < 50 y; 60.0 (35.3–81.2) ≥ 50 y	PPV 71.0%, NPV 92.2% <50 y; PPV 90.3%, NPV 36.0% ≥50 y	FP 12.5% (5/40) < 50 y; 36.4% (12/33) ≥ 50 y; FN NR	Partial verification; specificity/NPV poor in ≥ 50 y; not open screening
2	Singh et al., 2021 [27]	India	258	Thermalytix AI (non-contact IR; NIRAMAI)	Symptomatic women; >18 y (mean 41; range 18–80); 2 cancer centers, Bangalore	NR	Mammogram/US + biopsy histopathology; blinded independent interpretation	24.4% / 63 malignant, 195 benign	NR	Upper body precooled ~ 15 min; room 22–24 °C; 5 images from 5 angles; scan before standard imaging	Thermalytix AI; post-hoc cut-off 0.41 (Youden); pre-specified cut-off 0.5	82.5 (73.2–91.9)	80.5 (75.0–86.1)	PPV 57.8% (47.6–68.0); NPV 93.5% (89.7–97.2)	FN 11; FP 38	Post-hoc cut-off; partial verification; symptomatic spectrum bias
3	Da Luz et al. (2020) [25]	Brazil	25	Fluke Ti4000 (non-contact IR); SmartView 3.14	Women > 18 y after breast lump detection; mammography+biopsy within 3 months; no active tx	72% dense	Biopsy histopathology; mammography comparator only	40% (10 malignant, 15 benign)	Examples 30–32 mm; TNM NR	10-min acclimatisation; cold stress; images every minute for 15 min; t = 8 min analyzed	Custom thermal density analysis	83 (69–100)	74 (66–89)	PPV 62% (34–83); NPV 100% (89–100); κ = 0.54	NR / NR	Very small; dynamic protocol; enriched sample; conference paper
4	Alikhassi et al. (2018) [24]	Iran	156 breast-level cases (78 women)	Thermotekniks / ThermaGRAM^®^ (non-contact IR)	Women referred for breast US; same-day thermography; mean age 41.0 ± 10.4 y	NR	BIRADS ≥ 4 biopsy histopathology; BIRADS 1–3 1-year follow-up	4.5% / 7 malignant, 149 normal/benign	NR	Ambient acclimatisation; 5 images from 5 angles; room 24–26 °C; fixed distance	Certified medical thermographer; TH1/2 normal, TH3/4/5 suspicious	85.7	78.5	15.8% / 99.1%	FN 1; FP 32	Very low prevalence; partial verification; small malignant n
5	Sarigoz et al. (2018) [28]	Turkey	54	FLIR ThermaCam E45; Quick Report 1.2	Symptomatic women with single palpable lump; 18–70 y	NR	US-guided core biopsy histopathology; US/mammography ancillary	38.9% / 21 malignant, 33 benign	Median size 20 mm malignant vs. 18 mm benign; no TNM	15-min rest; room 22–25 °C; humidity 50%±15%; 2-m distance; 2 steady-state views	Human; ΔT symmetry analysis; positive if > 0.5 °C difference	95.24	72.73	PPV 60.61–100.00; NPV 95.24–100.00	NR / NR	Single-center symptomatic cohort; spectrum bias
6	Prasad et al. (2016) [29]	India	65	FLIR E60 and T650sc; Isotherm tool	Adults > 18 y with breast cancer confirmed on histo/cytopathology	NR	FNAC or biopsy histopathology	100% / 65 malignant	T1 11; T2 41; T3 9; T4 4	15-min rest after FNAC/biopsy; seated with hands raised; rotating chair; lateral/anterior/medial views; 0.5–1.0 °C isotherms	Manual expert review by 5 authors; no automation	92.31	—*	NR	FN 5; FP 5 contralateral	Cancer-enriched; specificity incalculable
7	Acharya et al. (2012) [30]	Singapore	50	NEC-Avio Thermo TVS2000 MkIIST; SVM CAD	25 normal + 25 cancerous thermograms from SGH	NR	Biopsy histopathology (image-level)	50% / 25 malignant, 25 normal	Stage III 15; Stage II 10; location: UOQ 50%, behind nipple 35%, UIQ 15%	1 m distance; room 20–22 °C; humidity 60%±5%; 15-min rest; loose gown; cycle days 5–12 and 21	Automatic SVM; texture features; 3-fold stratified CV	85.71	90.48	PPV 81.07; NPV NR	NR / NR	Small dataset; no external validation
8	Wishart et al. (2010) [26]	UK	100 women; 106 biopsies	Sentinel BreastScan / NoTouch BreastScan	Women undergoing breast core biopsy after abnormal exam or imaging	NR	Biopsy histopathology	61.3% (65 malignant, 41 benign)	62 invasive; 3 in situ; invasive grades I 13, II 35, III 14	Disrobed to waist; 5-min temperature-controlled airflow; 250 IR frames; blinded to biopsy	Multiple methods: Sentinel screening, Sentinel NN, expert review, NoTouch	78 expert; 70 NoTouch; 53 Sentinel screening; 48 Sentinel NN	NR expert; 75 NoTouch < 50 y; 48 NoTouch all ages; NR Sentinel	PPV 67; NPV 51 (NoTouch all)	NR / NR	Biopsy-referred; thermally silent cancers excluded; method-dependent performance
9	Parisky et al. (2003) [15]	USA	769 subjects; 875 lesions	BCS2100 computerized IR	Women with mammographically suspicious lesions recommended for biopsy; 5 centers	Reported as subgroup variable; denser tissue better specificity	Biopsy histopathology	21.4% / 187 malignant, 688 benign	NR; lesion-level; missed lesions mostly microcalcifications; size correlation in malignant masses	Prone bed; cooled-air chamber; sequential pre/during-cooling images; detailed settings NR	7 radiologists; 3 evaluators; mammogram-guided ROI; index-of-suspicion threshold	97 all lesions; 99 excl microcalcifications	14% (12–16%) overall; 18% (15–22%) excl microcalcifications	PPV 24% / NPV 95% overall; PPV 27% / NPV 99% excl microcalcifications	FN 13 / FP 1,544 overall; FN 1 / FP 768 excl microcalcifications	Post-mammography biopsy-referred; very low specificity; microcalcifications main FN source

*NR*  not reported, *IR*  infrared, *LCT*  liquid crystal thermography, *US*  ultrasound, *FNAC*  fine-needle aspiration cytology, *BIRADS*  Breast Imaging Reporting and Data System, *TNM*  tumor-node-metastasis, *SGH*  Singapore General Hospital

^*^Prasad et al. (2016) specificity was not calculable because the cohort was 100% cancer-enriched and contained no true-negative cases.

### Malaysian observational cohort

Table 4 presents the preliminary unpublished Malaysian cohort separately to preserve the distinction between peer-reviewed evidence and ongoing local data. In this cohort, 42 of 108 planned participants had been completed at the time of submission, with preliminary sensitivity of 72.2% and specificity of 58.3% based on the available cases.

**Table 4:** Preliminary unpublished data from ongoing malaysian cohort study (Universiti Sains Malaysia)

Field	Entry	Caveat / Uncertainty Measure
Study	Ainnur B et al., USM (Ongoing, Unpublished)	UNPUBLISHED — NOT PEER-REVIEWED. Must not be cited as evidence of LCT diagnostic performance.
Country	Malaysia	Single-centre; Universiti Sains Malaysia (USM)
Sample size	108 planned; 42 completed at submission	Highly preliminary. *n* = 42 is insufficient for precise or stable diagnostic performance estimates. Study is ongoing; results will change as enrolment continues.
Modality / Population	LCT Braster; women with dense breast tissue; initial screening	Inclusion/exclusion criteria: see study protocol. Reference standard: histopathology + imaging follow-up; not all cases biopsy-confirmed at this interim stage.
Sensitivity (preliminary)	72.2% (*n* = 42 completed cases)	HIGHLY IMPRECISE. No 95% CI calculable at this sample size. This figure will change substantially as enrolment proceeds to planned *n* = 108. Must not be compared with or combined with published estimates in Table 1.
Specificity (preliminary)	58.3% (*n* = 42 completed cases)	HIGHLY IMPRECISE. No 95% CI calculable at this sample size. Must not be compared with or combined with published estimates in Table 1.
Negative Predictive Value (NPV)	Cannot be calculated	NPV is prevalence-dependent and cannot be derived from sensitivity and specificity alone. No population cancer prevalence has been reported for this cohort. NPV must not be claimed, implied, or extrapolated from these preliminary data.
Key Limitation	Ongoing single-centre study; *n* = 42 of planned *n* = 108 completed; no 95% CI or FN/FP counts available; reference standard not uniformly biopsy-confirmed at this stage; no published peer-reviewed companion paper. These data are presented solely to describe the local Malaysian research context. They must not be used as evidence of LCT diagnostic performance or to support any clinical claims.	

These data are from an ongoing, single-centre cohort study at Universiti Sains Malaysia (USM). They are unpublished, not peer-reviewed, and are included solely to describe the local Malaysian research programme. They must not be interpreted as validated diagnostic performance data, compared with the published evidence in Table 3, or used to support any clinical claims regarding LCT accuracy. Final results will be reported upon study completion (planned *n* = 108) in a separate peer-reviewed publication

## Discussion

### Interpretation of diagnostic performance data

The nine studies summarised in Table 3 collectively illustrate the breadth and heterogeneity of the thermography evidence base rather than a coherent body of evidence supporting a specific level of diagnostic performance. Sensitivity across the included studies ranged from 78% [26] to 95.2% [28], and specificity from 14% [15] to 90.5% [30], but these figures cannot be pooled, ranked, or meaningfully averaged, as confirmed by the extreme between-study variability in specificity (I² = 99.1%) reported by Goni-Arana et al. [13]. The wide specificity range is particularly instructive. A specificity of 14% [15] means that approximately eight in ten disease-free women with mammographically suspicious lesions would receive an incorrect positive thermography result, a finding that generated 1,544 false-positive classifications across 688 benign lesions in a lesion-level analysis, partly attributable to the post-mammographic biopsy-referred design and the inherent inability of thermography to detect microcalcifications, which constituted the majority of false-negative findings in that study. At the other extreme, the 90.5% specificity figure [30] derives from a support vector machine model trained and tested on the same 50-patient dataset using three-fold cross-validation only, with no external validation cohort, making overfitting near-certain and the estimate ungeneralisable to any real-world screening population. The QUADAS-2 appraisal in Table 1 demonstrates that all nine studies carry high or unclear risk of bias in at least two of the four assessed domains, and all nine carry high risk in patient selection alone, meaning performance estimates are drawn from symptomatic, biopsy-referred, or cancer-enriched populations that systematically inflate apparent sensitivity and obscure the false-negative rates most relevant to screening contexts.

The only published prospective study using the Braster LCT device in a community screening population reported a sensitivity of 81.5% and specificity of 86.8% in women under 50 years of age; however, specificity fell substantially to 60.0%, and negative predictive value declined from 92.2% to 36.0% in women aged 50 years and over [17]. This age-stratified deterioration in specificity is clinically significant because women aged 50 and above represent the principal target population for breast cancer screening in most national programmes, including Malaysia [45]. Beyond the age-stratified finding, this study was conducted at a single centre, involved partial verification bias because BIRADS 1–2 cases were not biopsied, and did not fully stratify results by breast density subgroup. These limitations mean the figures cannot be accepted as established performance benchmarks and should be interpreted only as preliminary pilot data requiring prospective replication in adequately powered multicentre trials with pre-specified age-stratified and breast-density-stratified reporting endpoints.

The preliminary Malaysian cohort data (Table 4) — with sensitivity of 72.2% and specificity of 58.3% based on 42 completed cases of a planned 108 — are too imprecise to permit any performance conclusions and are not comparable to the published literature. No 95% confidence intervals are calculable at this sample size, the reference standard is not uniformly biopsy-confirmed at this interim stage, and the cohort remains incomplete. These data are reported solely to describe the local Malaysian research context and illustrate the clinical workflow; they must not be interpreted as evidence of LCT diagnostic performance or used to support any clinical claims regarding LCT accuracy in the Malaysian population.

### Clinical case illustrations: cohort workflow and classification failure scenarios

#### Concordant cases from the Malaysian cohort — workflow illustration

The following two cases are drawn from the ongoing single-centre Malaysian observational cohort (*n* = 42 completed at the time of writing). They are presented exclusively to illustrate the clinical workflow of Braster LCT in a real-world screening setting. These findings are preliminary, unpublished, and not peer-reviewed. They have not been subjected to formal statistical analysis, and the cohort is insufficiently powered to support any conclusions regarding diagnostic accuracy. These cases should not be interpreted as confirmatory evidence of LCT performance and are presented here solely to illustrate concordant classification scenarios observed within this cohort.

##### Case 1: true-positive — LCT concordant with confirmed malignancy

**Fig. 3:**
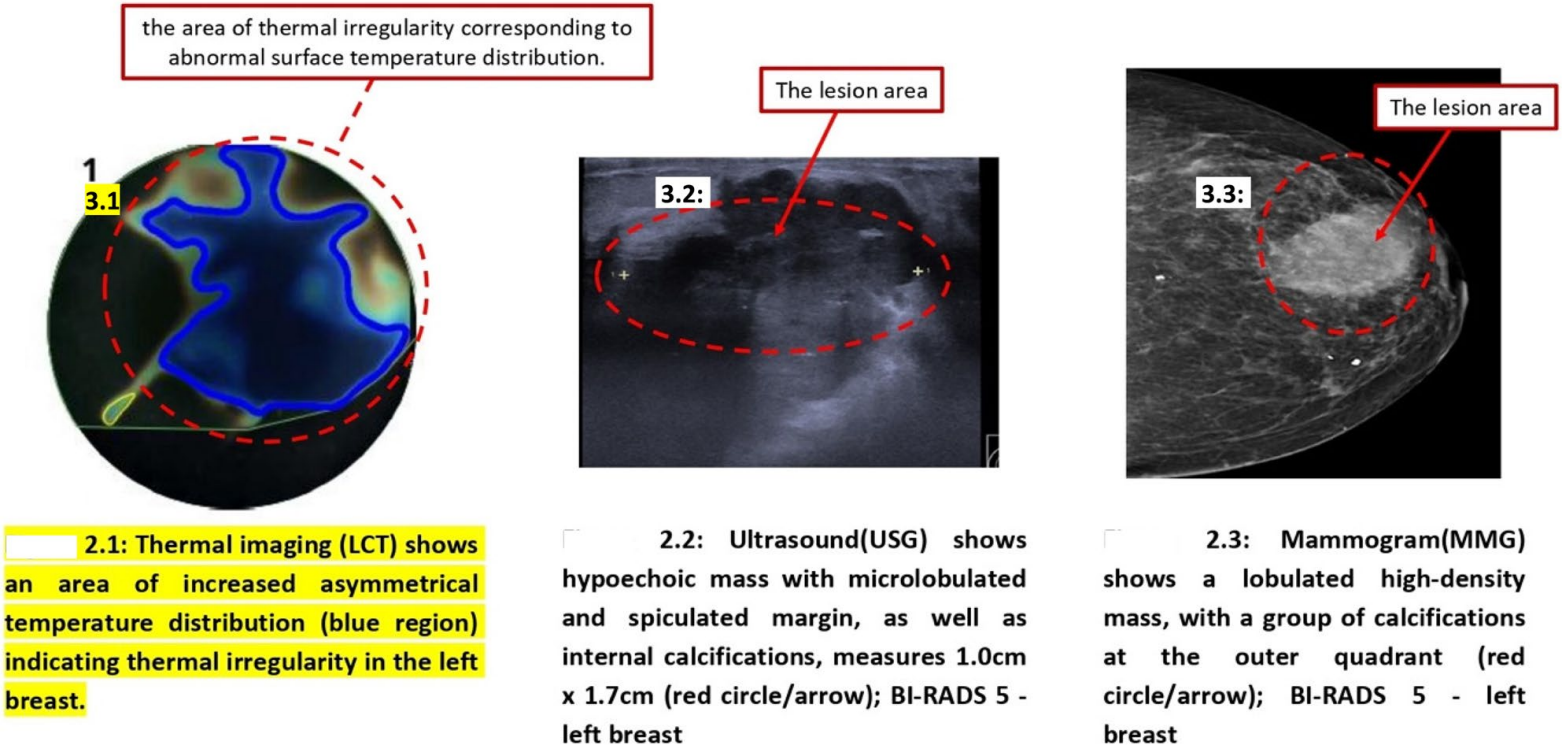
Representative LCT, ultrasonography, and mammographic findings from a true-positive case within the Malaysian cohort. LCT demonstrated left-sided thermal asymmetry concordant with biopsy-confirmed invasive breast carcinoma (no special type). This case contributes to the positive predictive value of LCT in the preliminary Malaysian cohort (*n* = 42). Data are unpublished and exploratory

A 59-year-old woman with a ten-year history of left breast swelling and heterogeneously dense breasts (ACR Type C) presented with a palpable mass. LCT demonstrated abnormal thermal asymmetry on the left side. Breast ultrasonography and mammography identified a suspicious mass, and histopathological examination confirmed invasive breast carcinoma, no special type. Blood investigations showed elevated fasting blood glucose (9.3 mmol/L) and fasting lipid profile (9.52 mmol/L), suggesting an associated metabolic risk profile. The patient was undergoing chemotherapy at the time of review. This case represents a true-positive LCT finding in a symptomatic, metabolically high-risk patient with heterogeneously dense breast tissue, and contributes to the positive predictive value of LCT within this preliminary cohort. Representative LCT, ultrasonography, and mammographic findings from this case are shown in Fig. 3.

##### Case 2: true-negative — LCT concordant with confirmed benign pathology

**Fig. 4:**
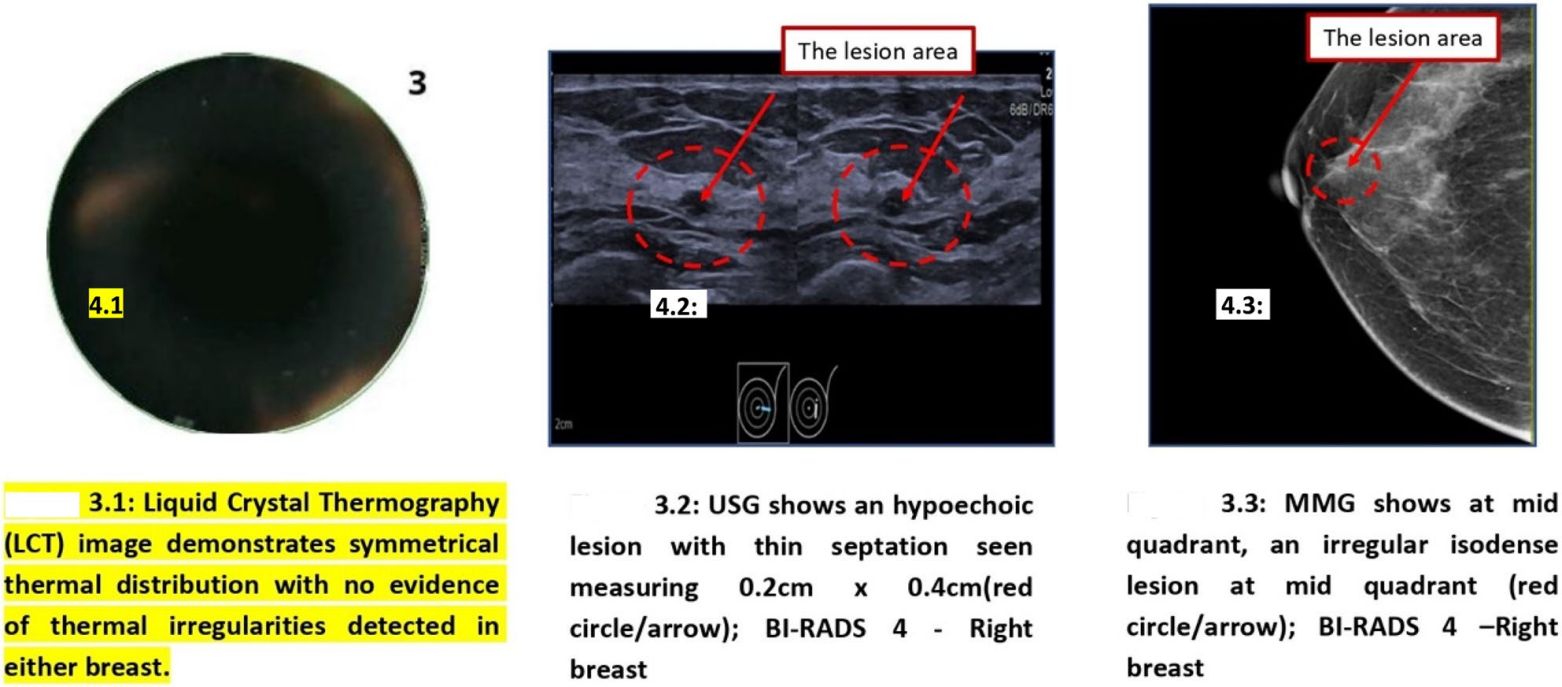
Representative LCT, ultrasonography, and mammographic findings from a true-negative case within the Malaysian cohort. LCT demonstrated bilaterally symmetrical thermal patterns concordant with biopsy-confirmed fibrocystic change (benign). Negative predictive value cannot be extrapolated from a single case. Data are unpublished and exploratory

A 50-year-old woman with a history of bilateral breast cysts underwent routine screening. Mammography and ultrasonography demonstrated BI-RADS 2–3 features consistent with benign pathology. LCT demonstrated bilaterally symmetrical and normal thermal patterns. Subsequent biopsy confirmed fibrocystic change, consistent with a true-negative diagnosis. Laboratory investigations showed normal fasting glucose (4.8 mmol/L) and mildly elevated lipids (5.71 mmol/L). This case represents a true-negative LCT finding in a woman with known benign breast disease, contributing to the negative predictive value of LCT in the preliminary cohort. It should be noted that negative predictive value is prevalence-dependent and cannot be extrapolated from this single case to a general screening population. Representative LCT, ultrasonography, and mammographic findings from this case are shown in Fig. 4.

#### Literature-derived illustrations of LCT misclassification failure modes

##### Case 3: false-positive — abnormal LCT with biopsy-confirmed benign pathology

A 48-year-old premenopausal woman with no family history of breast cancer and a prior clinical diagnosis of bilateral fibrocystic change presented for screening. LCT demonstrated focal thermal asymmetry in the right upper outer quadrant across Foil 2 and Foil 3 sequences, corresponding to a Zone 2 positive (abnormal) classification. Breast ultrasonography identified a 1.6 cm smooth-walled, anechoic lesion with no internal vascularity on Doppler assessment, consistent with a simple breast cyst. Digital mammography demonstrated no suspicious calcifications, architectural distortion, or density asymmetry (BI-RADS 2). Ultrasound-guided aspiration confirmed serous fluid; cytological examination was negative for malignancy. Final diagnosis: fibrocystic change with simple cyst, benign. LCT classification: false positive.

This case illustrates the inherent false-positive risk of contact LCT. Benign cystic change and fibrocystic tissue, which are common in premenopausal women, generate localised surface thermal asymmetry that is indistinguishable from malignancy-associated angiogenesis on LCT thermal mapping alone. An abnormal LCT finding in this context required structural imaging for clarification, representing an additional clinical step rather than any reduction in investigation burden. This is consistent with the known false-positive problem identified across the broader thermography literature, where benign inflammatory conditions including mastitis, fibrocystic change, and post-surgical change routinely produce false-positive thermal findings [15, 16].

##### Case 4: false-negative — normal LCT with biopsy-confirmed malignancy

A 56-year-old postmenopausal woman undergoing routine screening demonstrated bilaterally symmetrical and unremarkable thermal patterns on LCT across all three foil types, classified as Zone 0 (no thermal asymmetry detected). Concurrent standard digital mammography identified a 0.9 cm cluster of pleomorphic microcalcifications in the right upper outer quadrant, classified as BI-RADS 4 C. Stereotactic core biopsy confirmed intermediate-grade ductal carcinoma in situ (DCIS) without an invasive component. Final diagnosis: intermediate-grade DCIS, malignant. LCT classification: false-negative.

This case illustrates the principal false-negative limitation of LCT. DCIS without an associated invasive component generates insufficient angiogenic activity to produce detectable surface thermal asymmetry, and in this case, LCT provided no diagnostic contribution. Microcalcifications — the mammographic hallmark of DCIS — reflect structural mineralisation that is entirely beyond the detection capability of any thermographic approach. In this case, the malignancy would have been missed entirely if LCT had been used without concurrent mammography, providing a direct clinical illustration of why LCT cannot substitute for mammography in any screening context, consistent with the FDA position [21]. This finding is consistent with early liquid crystal contact thermography literature specifically identifying reduced detection for smaller lesions and non-angiogenic tumours as a persistent, mechanistically inherent limitation [19, 20], and is not unique to contact LCT; non-contact infrared thermography studies have also documented false-negative findings for non-angiogenic and low-grade in-situ disease, confirming that the limitation is mechanistically inherent to thermal surface assessment rather than to the specific acquisition [12, 19, 20].

##### Clinical misclassification analysis: net impact of LCT discordance

The four cases presented above together illustrate all three clinically important classification relationships, though they differ fundamentally in evidential status and must not be interpreted as equivalent. Cases 1 and 2 are observed findings from the Malaysian cohort, representing concordant true-positive and true-negative classifications in which LCT and standard imaging agreed. Cases 3 and 4 are literature-derived constructions representing the clinically more consequential discordant scenarios — false-positive and false-negative — that have not been observed in the current cohort and are presented solely to complete the mechanistic classification framework established in the preceding systematic evidence review.

In Case 3, LCT was false-positive while both mammography and ultrasonography correctly identified a benign finding: the net clinical impact of relying on LCT alone in this scenario would be unnecessary patient anxiety, additional imaging, and potentially unnecessary intervention. In Case 4, LCT was false-negative while mammography correctly identified a malignancy: the net clinical impact of relying on LCT alone would be a missed cancer. A fifth scenario — in which LCT is correct and mammography is false-negative, for example, in a deeply situated angiogenically active tumour in extremely dense tissue that is radiographically occult — has been hypothesised as a potential adjunctive role for LCT but was not observed in this preliminary cohort and has not been demonstrated in any published prospective study with consecutive enrolment and complete biopsy-based verification [13, 17].

The Malaysian cohort case series does not establish the frequency of any discordance pattern, as the cohort is too small and insufficiently powered for this analysis; the literature-derived scenarios presented in the preceding subsection represent mechanistic illustrations rather than frequency or prevalence data and cannot substitute for prospective observational evidence. A full discordance analysis with false-negative and false-positive counts stratified by breast density and menopausal status remains a prerequisite for any evidence-based positioning of LCT and must be a primary endpoint of any adequately powered prospective validation study [13, 22].

### LCT as an investigational adjunctive tool

On the basis of all evidence reviewed, LCT cannot be positioned as a primary screening tool, a standalone diagnostic tool, or a substitute for any established breast imaging modality. The FDA has explicitly stated that thermography should not be used in place of mammography to screen for or diagnose breast cancer [21], and that position is reflected consistently throughout this review. The only clinically realistic role consistent with the current evidence is as an investigational adjunctive or triage tool under evaluation — specifically, as a possible means of identifying women who may benefit from priority referral for further imaging in settings where standard imaging access is severely constrained. This is the context in which the Malaysian cohort described in this review was established, and it is precisely this adjunctive triage function, not replacement of mammography, that the ongoing study is designed to evaluate. Even this limited role has not been prospectively validated.

To reach the evidence standard required to support even an adjunctive clinical role, future research must demonstrate: (i) adequate sensitivity and specificity with 95% confidence intervals in a large prospective consecutive cohort with pre-specified enrolment criteria; (ii) false-negative and false-positive counts stratified by both breast density and menopausal status, given documented performance deterioration in postmenopausal women [17]; (iii) a head-to-head comparison against standard care with pre-specified misclassification endpoints; and (iv) net clinical benefit analysis accounting for the downstream consequences of both false-positive referrals and false-negative missed cancers. Until this evidence exists, LCT must be described and practised exclusively as an investigational research modality, deployed only within approved research protocols and interpreted only alongside — never in place of — standard imaging.

## Limitations

### Limitations of this review

This review was conducted as a narrative rather than a systematic review. No prospective protocol was registered, no PRISMA flow diagram was produced, and formal quantitative pooling of diagnostic performance estimates was not attempted. This choice reflects the extreme methodological heterogeneity of the thermography literature, including variation in device type, patient population, reference standard, acquisition protocol, and analytical approach, which makes meta-analytic pooling statistically unstable and difficult to interpret (I² = 99.1% for specificity across thermography studies) [13]. The resulting conclusions are therefore descriptive rather than formally pooled evidence synthesis. Only English-language publications were identified. The quality appraisal was completed by the review authors without independent external validation. Relevant studies may still have been missed despite the structured search approach used in the methods of this review.

### Limitations of the existing LCT evidence base

The published evidence base for Braster LCT specifically is narrow. It is dominated by one prospective pilot study Hodorowicz-Zaniewska et al., 2020; *n* = 274) [17], which was conducted at a single centre and had partial verification because not all participants underwent biopsy-based reference standard confirmation. The study also reported age-stratified performance, but breast-density stratification remained incomplete, and it did not include a concurrent head-to-head comparison against standard imaging with pre-specified misclassification endpoints. The remaining studies in the review are non-contact infrared thermography studies using different devices, populations, and reference standards, and therefore they are not directly comparable to Braster LCT as a device-specific evidence base.

Across the included studies, false-negative and false-positive counts stratified by breast density were not reported, so the evidence base does not support any meaningful subgroup-level inference for dense breasts. Confidence intervals for sensitivity and specificity are absent in many studies, limiting assessment of precision and making cross-study comparison difficult. Cancer prevalence is inconsistently reported, and stage or lesion size is often missing, which means predictive values vary substantially across studies and should not be extrapolated beyond the populations actually studied. In practical terms, these limitations mean that the available literature supports only cautious descriptive interpretation, not robust comparative claims about diagnostic performance in real-world screening settings.

### Braster-specific device limitations

The Braster contact LCT examination introduces device-specific and procedural limitations that are not present in non-contact infrared thermography and have not been fully characterised in the published literature [17, 23, 40]. Several issues are particularly important. Sequential application of multiple foils can introduce brief thermal perturbations at the skin surface, and differences in equilibration between applications may affect comparability across zones and examinations. Variations in foil pressure, placement angle, contact duration, and patient movement can all alter the recorded thermal pattern, yet these factors have not been standardised or reported with reproducibility data in published Braster studies. The handling of the foils is also important, because deformation, contamination, and static charge may affect colorimetric output, but published protocols do not address foil hygiene or reuse in detail.

Additional uncertainty comes from batch-to-batch variation in thermochromic foil behaviour, which could shift the temperature-colour response curve and alter the balance between false-positive and false-negative results. The output also depends on stable illumination, camera white balance, and ambient temperature, all of which can change the visible colour pattern without any true change in breast surface temperature. These limitations matter because they reduce reproducibility and make it harder to compare results across sites, operators, and study settings. They should therefore be addressed in any future standardisation work, multicentre validation study, or regulatory submission seeking to establish LCT as a validated clinical tool [13, 21].

## Future direction

The future clinical role of LCT, if any, will depend on the outcome of adequately powered prospective validation studies rather than on the extrapolation of current preliminary data. Dense-breast populations and low-resource settings with limited mammographic access represent the most clinically plausible contexts for future investigation of an adjunctive triage function, but this role has not yet been validated in any published prospective study with consecutive enrolment and complete biopsy-based verification. Advances in TLC materials, including improved microencapsulation techniques and tighter calibration tolerances, may improve the thermal sensitivity and reproducibility of contact LCT systems, but such technical improvements would still require clinical validation before any change in the evidence base could be claimed.

Machine learning and AI-assisted interpretation represent a possible direction for reducing operator-dependent variability in LCT image analysis and standardising classification thresholds, but these approaches have not yet been prospectively validated for LCT, and integration with telemedicine platforms remains at an early conceptual stage. Any future implementation pathway, whether in remote settings, dense-breast screening, or as a pre-referral triage tool, must be preceded by large-scale, prospective, multicentre clinical trials with pre-specified enrolment criteria, standardised acquisition protocols, and independent verification of false-negative and false-positive counts stratified by breast density and menopausal status. Until such evidence is available, LCT should not be positioned as ready for clinical translation and must remain within approved research frameworks.

## Conclusion

LCT is an investigational contact thermographic modality that may provide functional surface temperature information complementary to standard breast imaging, but it has not been validated as a standalone screening substitute for mammography or ultrasound. The FDA explicitly states that thermography should not replace mammography for breast cancer screening or diagnosis, and that position remains aligned with the current evidence base. In this review, LCT should therefore be understood only as a potential adjunctive research tool, not a clinical replacement for established imaging.

The published evidence base remains small and heterogeneous, with one prospective Braster pilot study and several non-comparable non-contact infrared thermography studies reporting widely variable performance estimates. Across these studies, heterogeneity is high, specificity varies markedly, and methodological differences limit any direct pooling or broad generalization. In addition, the illustrative false-negative case from the Malaysian cohort shows that DCIS can be missed by LCT even when mammography detects the lesion, reinforcing the mechanistic limitation that thermography cannot reliably detect microcalcifications or replace structural imaging. Until large, prospective, multicentre studies with consecutive enrolment, complete verification, and prespecified misclassification endpoints demonstrate consistent benefit, LCT should remain confined to approved research protocols and used only alongside standard imaging.

## Data Availability

No datasets were generated or analysed during the current study.
